# Rapid Quantification of Chlorpromazine Residues in Pork Using Nanosphere-Based Time-Resolved Fluorescence Immunoassay Analyzer

**DOI:** 10.1155/2021/6633016

**Published:** 2021-03-09

**Authors:** Wei Wang, Jingneng Wang, Min Wang, Juan Shen

**Affiliations:** ^1^Key Laboratory of Meat Processing and Quality Control, MOE, Key Laboratory of Meat Processing, MARA, Jiangsu Synergetic Innovation Center of Meat Processing and Quality Control, Nanjing Agricultural University, No. 1 Weigang, Nanjing, Jiangsu 210095, China; ^2^Shanghai Xiongtu Biotechnology Co., Ltd., No. 21 Guangfulin Road, Shanghai 201600, China

## Abstract

Immunochromatographic assays are good analytical tools for the detection of drug residues. We report a nanosphere-based time-resolved fluorescence immunoassay (nano-TRFIA) based on a monoclonal antibody and a portable TRFIA analyzer for the rapid quantification of chlorpromazine (CPZ) residues in pork. Under optimal conditions, the nano-TRFIA detected CPZ residues within 6 min of sample pretreatment. The results showed good linearity (*R*^2^ = 0.991), with a limit of detection (LOD) of 0.32 *μ*g/kg, a wide dynamic range of 0.46–10.0 *μ*g/kg, and coefficients of variation (CVs) of the overall intrabatch and interbatch assays of 7.34% and 7.65%, respectively. The nano-TRFIA was also used to detect CPZ at different spiked concentrations in pork, and the results were confirmed via ultraperformance liquid chromatography-tandem mass spectrometry (UPLC-MS/MS). The nano-TRFIA was evaluated for the analysis of six commercial pork samples, and the results agreed well with those obtained via UPLC-MS/MS, without significant differences (*P* > 0.05). Therefore, the proposed nano-TRFIA is a powerful alternative for the rapid and accurate quantification of CPZ residues in pork to meet the required Chinese maximum residue limits for veterinary drugs in foods.

## 1. Introduction

Chlorpromazine (CPZ) is a typical phenothiazine antipsychotic drug [1–3] and is common in clinical veterinary practice because of its strong sedative and antiemetic effects [4, 5]. The addition of CPZ into animal feeds causes sedation, hypnosis, weight gain, and fattening and shortens the slaughter time. Chlorpromazine can also attenuate the stress response and maintenance needs of animals and can decrease their weight loss and mortality during long-distance transportation and thus prevent the reduction of meat quality [6, 7]. However, CPZ residues in animal products can adversely affect human health [8–11]. As early as 1997, the European Union issued Commission Regulation (EC) No. 17/97 which banned the addition of CPZ into feeds. Japan has also stipulated that CPZ should not be present in foodstuffs of animal origin. According to the National Food Safety Standard on Maximum Residue Limits for Veterinary Drugs in Foods (GB31650-2019) in China, CPZ is allowed for treatment but should not be present in foods of animal origin. Nevertheless, some vendors still illegally add CPZ into edible animal feeds.

Detection methods for CPZ residues are underdeveloped compared with those for other antipsychotic drugs [12]. The CPZ residue detection methods can be classified into two types: the first type is based on chromatography, and it includes liquid microextraction-liquid chromatography [13], liquid chromatography-coulometry [14], high-performance liquid chromatography (HPLC) [15], liquid chromatography-mass spectrometry [16], and gas chromatography-mass spectrometry [17]. These methods require advanced laboratories, tedious sample pretreatment, long detection time, expensive apparatus, and operation by experienced personnel. Moreover, only a small number of samples can be analyzed at one time; therefore, these methods are not suitable for *in situ* detection or batch screening. The other type is based on immunological analyses, including enzyme-linked immunosorbent assay (ELISA), which is the most widely used. ELISA has been widely utilized to detect drug residues because of its high specificity, sensitivity, and simple operation [18–20]. However, this method suffers from low stability during quantification, low-temperature storage of reagents, short action time, long detection cycle, and unsuitability for testing a small number of samples. Thus, there is an urgent need for rapid, simple, convenient, and reliable detection methods.

Time-resolved fluorescence immunoassay (TRFIA) was developed in the early 1980s. This technology uses complexes of lanthanides and fluorescent markers and has been successfully applied to labeled immunoassays and clinical medicine [21, 22]. However, the detection sensitivity needs to be amplified through fluorescence enhancement due to the low fluorescence intensities of lanthanide complexes. Nanosphere-based TRFIA (nano-TRFIA) is a novel TRFIA that combines the long-lived fluorescence of rare-earth elements (Eu^3+^, Tb^3+^, etc.) with the signal amplification effect of nanospheres. Rare-earth elements and their complexes are codoped into nanospheres. After surface activation, an antibody is coupled onto the surface of a label to form a complex. During the immunoassay, this complex can elevate the sensitivity and expand the linear dynamic range [23]. In addition to facile operation, stable tracing, wide quantification, application ranges, and no radioactive contamination, nano-TRFIA can also offer higher sensitivity and accuracy than conventional methods; this is because polystyrene nanospheres containing thousands of lanthanide chelates have been developed as attractive labels with remarkable signal amplification potential [24]. Thus, in this study, we established a nano-TRFIA for the rapid quantification of CPZ residues in pork under the optimal experimental conditions described in our previous study [25]. Our goal is to provide references for quality and safety monitoring and the supervision and formulation of detection standards.

## 2. Materials and Methods

### 2.1. Materials and Reagents

Chlorpromazine, promethazine (PMZ), thioridazine (TDZ), acepromazine (ACP), prochlorperazine (PCZ), haloperidol (HAL), and fluphenazine (FPZ) were purchased from Dr. Ehrenstorfer Co. (Augsburg, Germany). Horseradish peroxidase-labeled goat anti-mouse immunoglobulin G (IgG) and tetramethylbenzidine (TMB) and stop buffer were obtained from Cell Signaling Technology (St. Louis, USA). Chlorpromazine-bovine serum albumin and mouse anti-CPZ monoclonal antibody were prepared in our laboratory [26]. Goat anti-mouse IgG was provided by Beijing Dingguo Changsheng Biotechnology Co., Ltd. (Beijing, China). Bovine serum albumin (BSA), 1-ethy1-3-(3-dimethylaminopropyl) carbodiimide hydrochloride (EDC), tert-butyl methyl ether (TBME), and MES buffer (0.05 M, pH 6.0) were purchased from Sigma-Aldrich (St. Louis, MO, USA). Europium-chelate-coated nanospheres (PS-COOH) with a size of 300 nm were synthesized and provided by Bangs Laboratories, Inc. (Fishers, IN, USA). Millipore 135 nitrocellulose (NC) membrane with a flow rate of 135 s/4 cm, conjugate pad, sample pad, and absorbent pad were obtained from Millipore (MA, USA). All solutions were prepared in deionized water (18.2 MΩ·cm, an Arium® Pro ultrapure water purification system from Sartorius, Göttingen, Germany). Two CPZ-positive pork samples labeled A and B were obtained from the Supervision, Inspection and Testing Center for Quality of Meat Products (Nanjing). Four pork samples labeled C, D, E, and F were acquired from a local supermarket in Nanjing, China. Other analytically pure reagents were obtained from Nanjing Chemical Reagent Co., Ltd. (Nanjing, China).

### 2.2. Detection of Cross-Reactivity of Monoclonal Antibody

The specificity of the antibody was determined via indirect competitive ELISA. Chlorpromazine, PMZ, TDZ, ACP, PCZ, HAL, and FPZ solutions at a series of concentrations were prepared and coated on 96-well microplates (Corning Inc., Corning, NY, USA). Mouse anti-CPZ monoclonal antibody was used as the primary antibody, and 0.01 M phosphate-buffered saline (PBS, pH 7.2) and CPZ were used as the negative and positive controls, respectively. Horseradish peroxidase-labeled goat anti-mouse IgG was used as the secondary antibody. After the antibody was incubated at room temperature for 1 h, washed, and mixed with TMB for color development, the optical density (OD) at 450 nm was measured using a Synergy H1 microplate reader (Bio-Tek, Winooski, VT, USA). The cross-reactivity (CR) was calculated as follows:

(1) $$\begin{array}{c} \text{CR}\left(\%\right)=\frac{{\text{IC}}_{50}\left(\text{CPZ}\right)}{{\text{IC}}_{50}\left(\text{CPZ structural analog}\right)}\mathrm{\times 100\%,} \end{array}$$

where IC_50_ is the half-maximal inhibitory concentration.

### 2.3. TRFIA Apparatus

The XT8201A portable TRFIA analyzer was codeveloped with Shanghai Xiongtu Biotechnology Co., Ltd., China, and used for the quantitative determination of CPZ. A light-emitting diode lamp served as the excitation source at 365 nm, and signals were acquired at 610 nm using a photomultiplier tube (PMT). The fluorescence signal was recorded using the PMT after a 400 *μ*s delay when the exciting light was irradiated on the test strip area. Meanwhile, the fluorescence with a short relaxation time decayed rapidly, reducing the background noise and achieving a high signal-to-noise ratio. The fluorescence signals on the test line (T line) and the control line (C line) peaks were processed using data processing software for quantitative analysis.

### 2.4. Labeling of Mouse Anti-CPZ Monoclonal Antibody by Fluorescent Nanospheres

Europium fluorescent nanospheres (100 *μ*L) were added to 500 *μ*L of 0.05 M boric acid buffer (pH 8.0). The mixture was vortexed and added to 100 *μ*L of 10 M EDC solution (prepared with 0.05 M MES buffer, pH 6.0). The obtained mixture was activated by shaking at room temperature for 30 min and centrifuged at 8000 r/min for 10 min to discard the supernatant. The precipitate was then redissolved with 500 *μ*L of 0.05 M boric acid buffer (pH 8.0), ultrasonicated for 5 min, and added to 100 *μ*L of mouse anti-CPZ monoclonal antibody. After the amount of fluorescent nanosphere-conjugated antibody was adjusted to 30 *μ*g/mL, the reaction was performed at room temperature for 2 h. Subsequently, 50 *μ*L of 0.05 M boric acid buffer (pH 8.0) containing 10% BSA was added, and the mixture was reacted at room temperature for 2 h on a thermomixer and centrifuged at 8000 r/min for 10 min to discard the supernatant. The precipitate was then redissolved with 500 *μ*L of 0.05 M boric acid buffer (pH 8.0) and ultrasonicated for 5 min. The labeled fluorescent nanospheres (1 : 800) were sprayed onto a bonding pad with a T2DDA film-spotting, gold-spraying instrument (Shanghai Hangan Electronic Technology Co., Ltd., Shanghai, China). After drying, the nanospheres were sealed and stored at room temperature prior to use.

### 2.5. Assembly of Nano-TRFIA Kit

**Figure 1:**
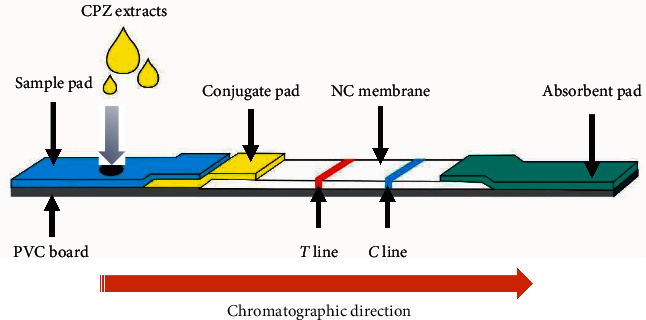
Description of the principle of nanosphere-based time-resolved fluorescence immunoassay (nano-TRFIA). NC: nitrocellulose; PVC: polyvinyl chloride; CPZ: chlorpromazine.

The assembly process of the nano-TRFIA kit is illustrated in Figure 1. First, 1.0 mg/mL CPZ-BSA and 1.0 mg/mL goat anti-mouse IgG were sprayed onto an NC membrane with a T2DDA film-spotting, gold-spraying instrument (Shanghai Hangan Electronic Technology Co., Ltd., Shanghai, China) as a T line and a C line, respectively (distance: about 1.0 cm), and dried overnight at 37°C. Afterward, the sample pad, conjugate pad, NC membrane, and absorbent pad were sequentially pasted on a polyvinyl chloride (PVC) board. The conjugate pad and sample pad were pasted at the T line. The conjugate pad and NC membrane overlapped by about 1-2 mm. The absorbent pad was pasted at the C line, and the pad overlapped with the NC membrane by about 1-2 mm. The PVC board was then cut into a 3.55 mm wide strip with a C6 strip-cutting machine (Shanghai Hangan Electronic Technology Co., Ltd., China) and placed into a plastic card to fabricate a nano-TRFIA kit and stored at room temperature for further experiments.

### 2.6. Sample Pretreatment for Nano-TRFIA

Pork tissue was homogenized at 10,000 r/min for 1 min, and 2.00 *g* (accurate to 0.01 *g*) was put into a 50 mL centrifuge tube with 500 *μ*L of 5 M NaOH solution and vortexed for 30 s. The sample was then mixed with 200 *μ*L of acetonitrile, vortexed for 80 s, and then shaken thoroughly for another 30 min. Next, 12 mL of TBME was added, vortexed for 80 s, and centrifuged at 4°C and 13,000 r/min for 15 min. Subsequently, the supernatant was blow-dried with nitrogen at 40°C, and the residue was redissolved with 2 mL of methanol. The solution was diluted with 0.01 M PBS (pH 7.2) and used for immediate measurement.

### 2.7. Nano-TRFIA for CPZ Detection

**Figure 2:**
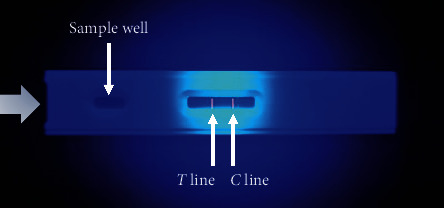
Image of chlorpromazine detection via nanosphere-based time-resolved fluorescence immunoassay under ultraviolet light.

All operations were performed at room temperature (22°C–28°C). First, 100 *μ*L of the extracted solutions described above was added into a sample well of the nano-TRFIA kit. After incubation at 37°C for 6 min, the nano-TRFIA kit was immediately inserted into the test strip slot of a portable TRFIA analyzer, and the fluorescence signals on the T line and C line were directly recorded under ultraviolet light (Figure 2).

### 2.8. Nano-TRFIA Standard Curve and Determination of Linearity

A CPZ standard sample was dissolved in methanol, calibrated, and mixed with a 1.0 g/kg standard solution. The system was then diluted to 10.0, 5.0, 2.0, 1.0, 0.5, 0.25, 0.125, 0.0625, and 0 *μ*g/kg solutions with 0.01 M PBS (pH 7.2). The CPZ standard solution was tested using the nano-TRFIA kit, and a portable TRFIA analyzer (Shanghai Xiongtu Biotechnology Co., Ltd., China) was used to record the fluorescence intensities of T and C lines and their ratios. Each standard was tested five times to plot the standard curve. The curve was drawn with LnX as the *x*-axis (where *X* is the concentration of competitive antigen), and the *B*/*B*_0_ ratio of the standard solution is seen at each concentration as the *y*-axis. The linear range was determined, where *B* is the *T*/*C* ratio after the addition of the CPZ standard solution and *B*_0_ is the ratio in the presence of 0 *μ*g/kg standard solution.

### 2.9. Determination of Limit of Detection and Limit of Quantification of Nano-TRFIA

Twenty blank samples were randomly taken for the nano-TRFIA strip test to calculate the mass concentration of the blank sample. The average (*X*) and standard deviation (SD) were obtained. The limit of detection (LOD) and limit of quantification (LOQ) were calculated according to LOD = *X* + 3SD and LOQ = *X* + 10SD, respectively.

### 2.10. Detection of Accuracy and Reproducibility of Nano-TRFIA

The accuracy and reproducibility of this method were represented by intrabatch and interbatch coefficients of variation (CVs). Chlorpromazine standard solutions at three different concentrations were tested. Two batches of the same sample were tested, and each batch was detected 10 times. The average of three CVs for each batch was employed as the intrabatch CV, and the average of two intrabatch CVs was used as the overall CV. The interbatch CV was calculated by measuring the CVs of two batches at each concentration 20 times and then averaging the values.

### 2.11. Comparison of Nano-TRFIA and Ultraperformance Liquid Chromatography-Tandem Mass Spectrometry Results

To validate the nano-TRFIA results, six negative minced pork samples were treated with CPZ standard solutions at 1.0, 2.0, 4.0, 6.0, 8.0, and 10.0 *μ*g/kg and then subjected to ultraperformance liquid chromatography-tandem mass spectrometry (UPLC-MS/MS) (Waters Corp., Milford, MA, USA) [16, 27]. The spiked samples were pretreated with acetonitrile to precipitate proteins, followed by extraction with TBME. Separation was performed on a Waters ACQUITY UPLC BEH C18 column (50 mm × 2.1 mm, 1.7 *μ*m). The column temperature was 30°C, and the sample injection volume was 10.0 *μ*L. Gradient elution was performed with solutions A (acetonitrile) and B (0.1% (*v*/*v*) formic acid in water) as the mobile phases, at a flow rate of 0.3 mL/min: 0–2 min, 10% A and 90% B; 2–4 min, 60% A and 40% B; and 4-5 min, 10% A and 90% B [25]. Detection was performed via positive-ion electrospray ionization in multiple reaction monitoring mode. The transition of *m*/*z* 319.27–85.96 was used to quantify CPZ. The recovery rate and relative standard deviation (RSD) of the samples were calculated.

### 2.12. Application in Real Pork Sample

To validate our strategy, six pork samples from different products were used to detect CPZ. Chlorpromazine from pork samples was extracted as described, and the extracts were analyzed through both nano-TRFIA and UPLC-MS/MS.

## 3. Results and Discussion

### 3.1. CR of Monoclonal Antibody

Indirect competitive ELISA was conducted to detect the CRs between mouse anti-CPZ monoclonal antibody and other six structurally relevant compounds. As presented in Table 1, the antibody had an excellent specificity (CR: <0.77%), with mild reactivity with only PCZ (CR: 5.74%) among the considered chemicals. Therefore, there was either low or no CR during CPZ detection, which ensures the specificity and accuracy of the proposed nano-TRFIA. We postulate that the stronger binding of the antibody to PCZ than to the other chemicals is because the key structure of PCZ is similar to that of CPZ.

### 3.2. Standard Curve and Linearity of Nano-TRFIA

**Figure 3:**
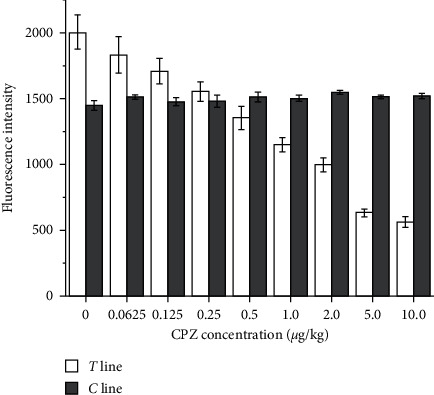
Fluorescence intensity of nanosphere-based time-resolved fluorescence immunoassay with 0, 0.0625, 0.125, 0.25, 0.5, 1.0, 2.0, 5.0, and 10.0 *μ*g/kg of chlorpromazine (CPZ). The *x*-axis is the fluorescence intensity on the T line and C line peaks at different CPZ concentrations; the *y*-axis is a standard solution at different concentrations.

**Figure 4:**
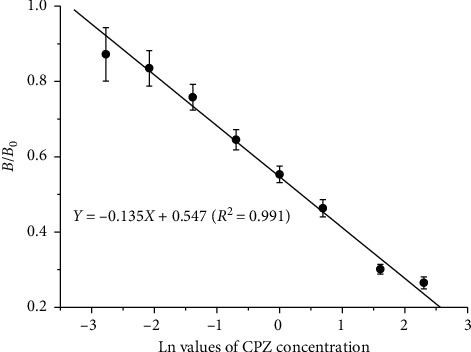
Standard curve of chlorpromazine (CPZ) detection via nanosphere-based time-resolved fluorescence immunoassay (eight levels of serial dilution containing 0.0625, 0.125, 0.25, 0.5, 1.0, 2.0, 5.0, and 10.0 *μ*g/kg of CPZ were prepared in PBS). The *x*-axis is the Ln value of CPZ concentration; the *y*-axis is the *B*/*B*_0_ ratio of standard solution at each concentration.

To establish a standard curve, the fluorescence intensities on the T line and the C line with different CPZ concentrations were recorded using a portable TRFIA analyzer (Figure 3). As shown in Figure 4, the standard curve was plotted with the logarithm of the concentration of the competitive antigen CPZ standard as the *x*-axis and the *B*/*B*_0_ ratio as the *y*-axis. The curve shows good linearity when the logarithm ranges from −2.773 to 2.303, corresponding to concentrations of 0.0625 to 10.0 *μ*g/kg. The fitted linear regression equation is *Y* = −0.135*X* + 0.547 (*R*^2^ = 0.991), meeting the requirement for a linear relationship.

### 3.3. LOD and LOQ of Nano-TRFIA

The *B*/*B*_0_ values measured with 20 blank samples were substituted into the above linear regression equation, giving an average CPZ concentration of 0.26 *μ*g/kg and an SD of 0.02. According to the equations $\text{LOD}=\bar{X}+3\text{SD}$ and $\text{LOQ}=\bar{X}+10\text{SD}$, the LOD and LOQ were calculated as 0.32 *μ*g/kg and 0.46 *μ*g/kg, respectively. The established nano-TRFIA thus had a much higher sensitivity than those previously reported [7, 17, 26, 28, 29]. This suggests that the nano-TRFIA has practical application value.

### 3.4. Accuracy and Reproducibility of Nano-TRFIA

The accuracy of the nano-TRFIA was determined by setting three concentrations of CPZ standard solutions (1.0, 5.0, and 10.0 *μ*g/kg), dividing the samples into two batches, and repeatedly testing each batch 10 times (Table 2). The intrabatch CVs of the first and second batches were 6.32% and 8.36%, respectively. The overall intrabatch and interbatch CVs were 7.34% and 7.65%, respectively. This method is highly accurate and repeatable since all CVs were lower than 10.0%.

### 3.5. Validation by UPLC-MS/MS

To verify the accuracy of this method, the correlation of the measurement results with those of UPLC-MS/MS was analyzed. For UPLC-MS/MS, the standard curve was plotted via CPZ standard solutions at mass concentrations of 0.25, 0.5, 1.0, 2.5, 5.0, and 10.0 *μ*g/kg, with a regression equation of *Y* = 11028.80*X* − 1403.87 (*R*^2^ = 0.994). The retention time of CPZ was 2.2 min. The LOD was 0.25 *μ*g/kg, and the LOQ was 1.0 *μ*g/kg.

**Figure 5:**
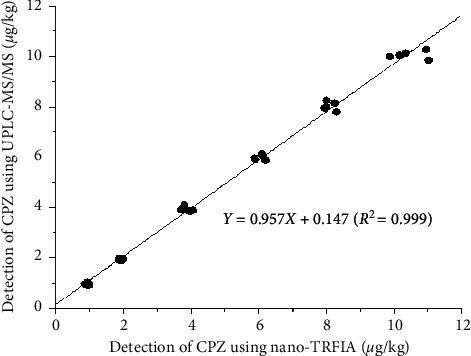
Correlation analysis of nanosphere-based time-resolved fluorescence immunoassay (nano-TRFIA) and ultraperformance liquid chromatography-tandem mass spectrometry (UPLC-MS/MS) results (six negative minced pork samples were added to chlorpromazine (CPZ) standard solutions at 1.0, 2.0, 4.0, 6.0, 8.0, and 10.0 *μ*g/kg). The *x*-axis is the detection result of CPZ using nano-TRFIA; the *y*-axis is the detection result of CPZ using UPLC-MS/MS.

Samples were tested via nano-TRFIA and UPLC-MS/MS under the same spiked concentration (Table 3). The results were subjected to linear regression analysis, giving an equation of *Y* = 0.957*X* + 0.147 (*R*^2^ = 0.999) (Figure 5). The CPZ in the samples could be accurately and reliably detected via the established nano-TRFIA.

### 3.6. Determination and Evaluation of Real Pork Samples

To examine the applicability of the established nano-TRFIA, we selected six different pork samples and compared the results with those of nano-TRFIA and UPLC-MS/MS (Table 4). The results obtained using the nano-TRFIA kits were nearly consistent with the UPLC-MS/MS detection results. There was no significant difference between the results of the two methods (*P* > 0.05). The data could be used to evaluate the applicability and reliability of the newly developed method. Thus, this nano-TRFIA is a potential alternative to chromatography for the regulatory analysis of CPZ residues in foodstuffs of animal origin.

## 4. Conclusion

Detecting CPZ residues in meat is important; hence, several countries have formulated standards for CPZ residues. Although China has continuously focused on the monitoring of CPZ during food safety inspections and has enforced a strict ban on the addition of CPZ to animal feed, CPZ residues have still been detected in pork in recent years. The strategy reported in this study combines nano-TRFIA with immunochromatography to realize high specificity, sensitivity, reproducibility, and a wide linear range. The detection results meet the requirements for quantifying actual samples. This method is readily operable and does not require expensive instrumentation. Moreover, the approach can quickly measure CPZ residues in foodstuffs of animal origin *in situ* and may have application value for other drug residues.

## Figures and Tables

**Table 1:** CR of mouse anti-CPZ monoclonal antibody.

Standard	IC_50_ (*μ*g/kg)	CR (%)
CPZ	15.38	100
PCZ	267.90	5.74
ACP	>2000	<0.77
TDZ	>2000	<0.77
PMZ	>2000	<0.77
HAL	>2000	<0.77
FPZ	>2000	<0.77

CR, cross-reactivity; ACP, acepromazine; CPZ, chlorpromazine; FPZ, fluphenazine; HAL, haloperidol; IC_50_, half-maximal inhibitory concentration; PCZ, prochlorperazine; PMZ, promethazine; TDZ, thioridazine.

**Table 2:** CPZ detection by nano-TRFIA.

CPZ concentration (*μ*g/kg)	First batch (*n* = 10)			Second batch (*n* = 10)		
	Mean value (*μ*g/kg)	SD	CV (%)	Mean value (*μ*g/kg)	SD	CV (%)
1.0	0.97	0.02	2.61	0.95	0.06	8.51
5.0	4.86	0.04	7.82	5.04	0.04	8.85
10.0	11.87	0.03	8.53	11.31	0.02	7.71

CPZ, chlorpromazine; CV, coefficient of variation; nano-TRFIA, nanosphere-based time-resolved fluorescence immunoassay; SD, standard deviation.

**Table 3:** Comparison of nano-TRFIA and UPLC-MS/MS for CPZ detection in pork (*n* = 5).

Spiked concentration (*μ*g/kg)	UPLC-MS/MS			Nano-TRFIA		
	Detected concentration (*μ*g/kg)	Recovery (%)	RSD (%)	Detected concentration (*μ*g/kg)	Recovery (%)	RSD (%)
1.0	0.96 ± 0.03	96.40	3.99	0.93 ± 0.05	92.60	5.59
2.0	1.92 ± 0.04	96.20	2.25	1.92 ± 0.04	95.80	2.11
4.0	3.92 ± 0.09	98.00	2.63	3.89 ± 0.12	97.15	3.59
6.0	5.98 ± 0.10	99.73	1.80	6.04 ± 0.13	100.63	2.40
8.0	8.03 ± 0.16	100.35	2.23	8.09 ± 0.15	101.15	2.09
10.0	10.06 ± 0.14	100.58	1.56	10.47 ± 0.45	104.70	4.77

CPZ, chlorpromazine; nano-TRFIA, nanosphere-based time-resolved fluorescence immunoassay; RSD, relative standard deviation; UPLC-MS/MS, ultraperformance liquid chromatography-tandem mass spectrometry.

**Table 4:** Comparison of nano-TRFIA and UPLC-MS/MS for CPZ detection in real pork samples (*n* = 5).

Sample	CPZ found (*μ*g/kg)	
	UPLC-MS/MS	Nano-TRFIA
A	1.62 ± 0.07	1.57 ± 0.06
B	1.95 ± 0.09	1.92 ± 0.05
C	Nd	Nd
D	Nd	Nd
E	Nd	Nd
F	Nd	Nd

CPZ, chlorpromazine; nano-TRFIA, nanosphere-based time-resolved fluorescence immunoassay; UPLC-MS/MS, ultraperformance liquid chromatography-tandem mass spectrometry; nd, not detected.

## Data Availability

The original data used to support the findings of this study can be obtained from the corresponding author upon request.
